# Supplementary material for: lncRNA MELTF-AS1 facilitates osteosarcoma metastasis by modulating MMP14 expression
Source: Mol Ther Nucleic Acids. 2021 Aug 26;26:787–97. doi: 10.1016/j.omtn.2021.08.022 (PMC8526484; doi:10.1016/j.omtn.2021.08.022)
Supplement: Document S1. Figures S1 and S2 and Table S1 [file mmc1.pdf]

OMTN, Volume *26*

Supplemental information

lncRNA MELTF-AS1 facilitates osteosarcoma metastasis by modulating MMP14 expression

Lei Ding, Taiyuan Liu, Yuan Qu, Zhichen Kang, Lixin Guo, Haina Zhang, Junjie Jiang, Fuling Qu, Wanbao Ge, and Shanyong Zhang

Supplemental Information

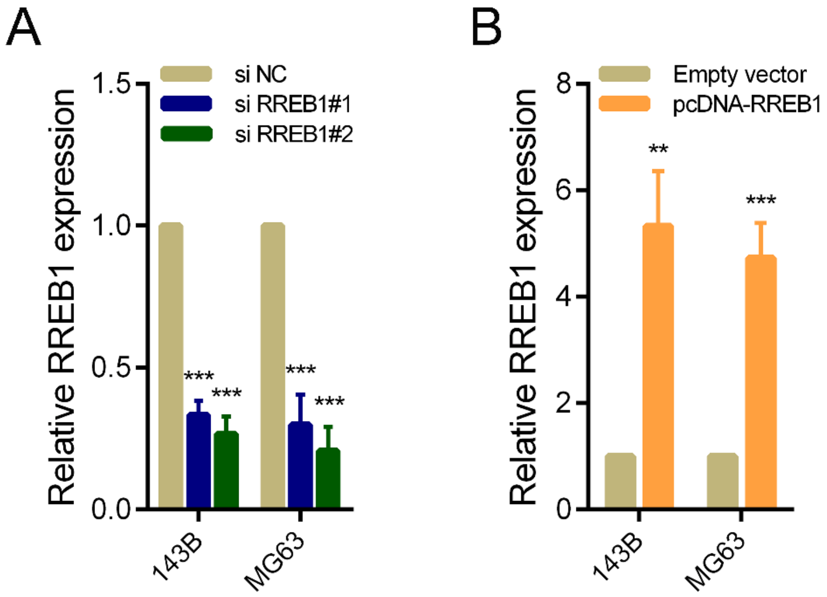

Figure S1. (A) QPCR analysis of RREB1 expression after knockdown of RREB1 in osteosarcoma cells. **(B)** QPCR analysis of RREB1 expression after overexpressing RREB1 in osteosarcoma cells. **P < 0.01 and ***P < 0.001.

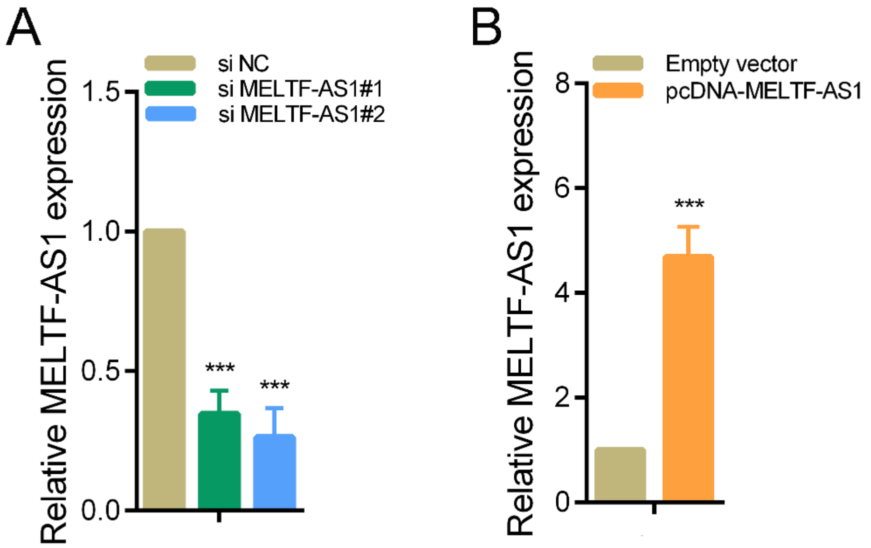

Figure S2. (A) QPCR analysis of MELTF-AS1 expression after knockdown of MELTF-AS1 in 143B cells. **(B)** QPCR analysis of MELTF-AS1 expression after overexpressing MELTF-AS1 in 143B cells. ***P < 0.001.

Table S1: The probability of each transcription factor binding to the MELTF-AS1 promoter region predicted by the JASPAR tool, the higher the score, the greater the probability.

Transcription factor	Score	Start	End	Strand	predicted site sequence
RREB1	18.844	312	331	-1	CCCCACACCACCACCCCTCG
Zfx	16.178	1491	1504	-1	CCGGCCGGGGCCTC
E2F3	16.047	1977	1991	1	CTCCCGCCGCCGCCA
PPARG::RXRA	16.039	548	562	1	GTGGGGCAGGGGGCA
TP63	15.596	856	875	1	CTGCATGTGCTGGCTTGTTT
EGR2	15.411	1721	1735	-1	CCCGCACCCACGCCC
Spi1	15.367	1404	1418	-1	ATGAAGAGGAAGAGA
Zfx	15.141	1867	1880	1	CCGGGCTCGGCCTC
EGR1	15.113	1977	1990	1	CTCCCGCCGCCGCC
SP2	14.648	1522	1536	-1	TGCCCGCCCCTCCGC
EGR1	14.636	413	426	1	GGCCCACCCCCACC
ZNF263	14.560	1394	1414	-1	AGAGGAAGAGAAGAAATGGGA
E2F3	14.381	1973	1987	1	CTCCCTCCCGCCGCC
Myod1	14.187	631	643	-1	TCCAGCTGCCTCC
Nkx2-5 (var.2)	14.170	795	805	-1	AGGCACTCAAA
HSF1	13.960	1437	1451	1	GTTCTGGAAACTTTT
ZNF263	13.959	1400	1420	-1	AAATGAAGAGGAAGAGAAGAA
E2F4	13.950	1975	1985	-1	CGGCGGGAGGG
RREB1	13.844	313	332	-1	CCCCCACACCACCACCCCTC
TP53	13.843	849	863	-1	ACATGCAGAGGCTTG
E2F6	13.669	1876	1886	-1	GGGAGGGAGGC
E2F6	13.669	1971	1981	-1	GGGAGGGAGGC
PRDM1	13.636	86	100	-1	TGGAAAGGAAAGTTA
KLF5	13.540	1915	1924	1	CCCCCACCCC
Zfx	13.392	1657	1670	1	GAGCCCGCGGCCTG
STAT3	13.347	94	104	-1	CTTCTGGAAAG
STAT2::STAT1	13.336	86	100	1	TAACTTTCCTTTCCA
Myog	13.329	634	644	1	GGCAGCTGGAA
TEAD1	13.305	637	648	-1	CACATTCCAGCT
POU2F2	13.293	1337	1349	-1	GCCATTTGCATTC
RFX2	13.191	1196	1214	1	GGTTGCATGGCTACAGCCG
TP53	13.147	858	872	1	GCATGTGCTGGCTTG
INSM1	13.033	551	562	1	GGGCAGGGGGCA
SP2	13.021	413	427	1	GGCCCACCCCCACCT
Tcf12	12.992	634	644	1	GGCAGCTGGAA
Zfx	12.906	1962	1975	1	CCCGGTCCGGCCTC
Bach1::Mafk	12.873	912	926	1	GCACTGACACAGCAG
Nfe2l2	12.862	911	925	1	AGCACTGACACAGCA
Spi1	12.795	108	122	1	AGGGTAGGGAAGTGG
ZEB1	12.786	1274	1282	-1	CCCCACCTG
NR2C2	12.757	548	562	1	GTGGGGCAGGGGGCA
ZNF263	12.735	550	570	1	GGGGCAGGGGGCAGCAGAGAA
Erg	12.604	1404	1414	-1	AGAGGAAGAGA
EGR2	12.599	1350	1364	1	TCCGCGCCCACGCGG
Nr1h3::Rxra	12.591	75	93	1	TGCCCAAAGGTTAACTTTC
Tcf3	12.445	148	158	-1	CTCAGCTGCAC
ESR1	12.424	1170	1189	-1	AGTCCCGGGCACCTTCACCC
SP1	12.418	413	423	1	GGCCCACCCCC
EGR2	12.410	413	427	1	GGCCCACCCCCACCT
INSM1	12.373	938	949	-1	TGTCAGGTGGCA
EGR2	12.343	966	980	1	ACTGCACCCACGCAG
STAT5	12.339	248	258	-1	TTTTCCATGAA

**ZNF263**	12.296	1397	1417	-1	TGAAGAGGAAGAGAAGAAATG
**E2F6**	12.239	349	359	1	GAGAGGGAAGC
**Klf1**	12.225	980	990	1	GGCCACACCTT
**Zfx**	12.178	1139	1152	-1	AGGACCTGGGCCTC
**KLF5**	12.124	477	486	1	CCCACGCCCC
**STAT3**	11.996	247	257	1	CTTCATGGAAA
**Sox3**	11.991	1120	1129	-1	GCTTTGTCCC
**TLX1::NFIC**	11.937	1346	1359	1	TGGCTCCGCGCCCA
**SP1**	11.891	1526	1536	-1	TGCCCGCCCCT
**Tcf3**	11.821	633	643	-1	TCCAGCTGCCT
**YY1**	11.821	1475	1486	1	CAAGATGGAGTA
**RUNX2**	11.813	433	447	-1	GTGCTCTGTGGTATC
**RUNX1**	11.773	434	444	-1	CTCTGTGGTAT
**RREB1**	11.722	315	334	-1	GGCCCCCACACCACCACCCC
**EGR2**	11.693	1723	1737	-1	CGCCCGCACCCACGC
**Nfe2l2**	11.682	489	503	1	TCCAGTGAGTTAGCA
**SP1**	11.679	1915	1925	1	CCCCCACCCCG
**Nr2e3**	11.665	1126	1132	-1	CAAGCTT
**E2F1**	11.654	1706	1716	-1	CTGGCGGCAGC
**SREBF2**	11.652	1167	1176	1	GTGGGGTGAA
**E2F6**	11.620	1975	1985	-1	CGGCGGGAGGG
**Sox6**	11.606	1120	1129	-1	GCTTTGTCCC
**Spi1**	11.597	1396	1410	-1	GAAGAGAAGAAATGG
**TP63**	11.594	846	865	-1	GCACATGCAGAGGCTTGGAC
**ELF1**	11.542	1405	1417	-1	TGAAGAGGAAGAG
**EGR1**	11.525	1276	1289	-1	GGCTCGCCCCCACC
**Zfx**	11.509	1219	1232	1	GCTGCCAGGGCCTC
**Klf4**	11.453	1915	1924	-1	GGGGTGGGGG
**PLAG1**	11.434	63	76	1	GAGGGGCTAGGGTG
**RORA_2**	11.416	180	193	1	ACAAAGTCGGTCAA
**TP53**	11.401	859	873	-1	ACAAGCCAGCACATG
**Tcfcp2l1**	11.364	213	226	-1	CCAGTCAATTCCTG
**NFATC2**	11.360	252	258	-1	TTTTCCA
**Tcfcp2l1**	11.316	665	678	1	CCTGCAAGAGCCAG
**SP2**	11.286	1840	1854	-1	GCCCTGCCTCGTCCC
**Ddit3::Cebpa**	11.285	1194	1205	-1	CCATGCAACCCC
**Nfe2l2**	11.279	138	152	1	TGGAGTGACTGTGCA
**NHLH1**	11.214	148	159	-1	TCTCAGCTGCAC
**Klf4**	11.207	477	486	-1	GGGGCGTGGG
**SREBF1**	11.106	1167	1176	-1	TTCACCCCAC
**Tcfcp2l1**	11.099	1812	1825	-1	CCAGCTCCATCCCG
**ESR2**	11.073	1664	1678	-1	AGGTGACCCAGGCCG
**Rfx1**	11.052	1196	1209	1	GGTTGCATGGCTAC
**Zfx**	11.052	1555	1568	-1	ACCCGCTGGGCCTG
**EGR2**	11.024	1561	1575	-1	GCCCCGCACCCGCTG
**USF2**	10.886	938	948	-1	GTCAGGTGGCA
**EGR1**	10.878	1980	1993	1	CCGCCGCCGCCAGC
**Myc**	10.867	858	867	1	GCATGTGCTG
