# Supplementary material for: lncRNA MELTF-AS1 facilitates osteosarcoma metastasis by modulating MMP14 expression
Source: Mol Ther Nucleic Acids. 2021 Aug 26;26:787–97. doi: 10.1016/j.omtn.2021.08.022 (PMC8526484; doi:10.1016/j.omtn.2021.08.022)
Supplement: Document S2. Article plus supplemental information [file mmc2.pdf]

Molecular Therapy
Nucleic Acids
Original Article

lncRNA MELTF-AS1 facilitates osteosarcoma metastasis by modulating MMP14 expression

Lei Ding,[1,5] Taiyuan Liu,[2,3,5] Yuan Qu,[4] Zhichen Kang,[1] Lixin Guo,[1] Haina Zhang,[1] Junjie Jiang,[1] Fuling Qu,[1] Wanbao Ge,[4] and Shanyong Zhang[4]

[1]Department of Rehabilitation, The Second Hospital of Jilin University, Changchun 130000, Jilin, China; [2]Department of Breast Surgery, The Second Hospital of Jilin University, Changchun 130000, Jilin, China; [3]Department of Breast Surgery, The Second Affiliated Hospital of Dalian Medical University, Dalian 116023, Liaoning, China; [4]Department of Spine Surgery, The Second Hospital of Jilin University, Changchun 130000, Jilin, China

Osteosarcoma is a highly aggressive cancer common in children and adolescents. There is still a lack of effective treatments for metastatic or recurrent osteosarcoma. The role of long non-coding RNAs (lncRNAs) in osteosarcoma has gradually attracted attention. Here, we identified lncRNAs that were abnormally expressed in metastatic osteosarcoma through analyzing the sequencing data of osteosarcoma tissues and selected upregulated lncRNA MELTF-AS1 for detailed study. The qRT-PCR analysis showed that the expression of MELTF-AS1 was increased in osteosarcoma tissues and cells, and the high expression of MELTF-AS1 indicated a poor prognosis of osteosarcoma patients. The high expression of MELTF-AS1 in osteosarcoma was partly due to the transcriptional activation of RREB1. The results of transwell assays, scratch wound healing assays, and the tail vein injection lung metastasis model demonstrated that knocking down MELTF-AS1 inhibited metastasis ability of osteosarcoma cells. Furthermore, the results of RNA pull-down assays, luciferase reporter assays, and RNA immunoprecipitation (RIP) assays revealed that MELTF-AS1 could regulate MMP14 expression through interaction with miR-485-5p. Our study suggested that MELTF-AS1 functioned as a pro-metastasis gene in osteosarcoma by upregulating MMP14 and that it could be a potential therapeutic and diagnostic target for osteosarcoma.

INTRODUCTION

Osteosarcoma is the most common primary malignant bone tumor in children and adolescents arising from primitive transformed cells of mesenchymal origin.[1] With the advancement of diagnostic technology and treatment strategies, the overall prognosis of patients with osteosarcoma has been greatly improved. However, for osteosarcoma with distant metastasis, surgical resection and traditional chemotherapy cannot improve outcomes. Effective treatment for patients with metastatic or recurrent osteosarcoma remains a significant clinical challenge.[2–4] Clarifying the mechanism of osteosarcoma metastasis and finding novel therapeutic targets have become key issues in osteosarcoma research.

With the development of high-throughput sequencing technology, many studies have revealed that the proportion of non-coding RNAs (ncRNAs) in the human genome accounts for more than 95%.[5] As one of the important components of ncRNA, long non-coding RNAs (lncRNAs) have attracted a lot of attention. Although they lack the ability to encode proteins, they are widely involved in regulation of gene expression. lncRNAs have been found to play vital roles in important biological processes such as chromatin modification, gene transcription, and mRNA degradation and translation, and their variation and abnormal expression can affect multiple diseases, including tumors.[6–8] Some lncRNAs have been reported to be involved in the proliferation, metastasis, and drug resistance of osteosarcoma, but the role of most lncRNAs in osteosarcoma remains unclear.[9]

In this study, we first identified the abnormally expressed lncRNAs in osteosarcoma tissues with distant metastasis by analyzing high-throughput sequencing data. These lncRNAs presumably play important roles in osteosarcoma metastasis. From these metastasis-related lncRNAs, we selected MELTF-AS1 (also known as MFI2-AS1) for further research because of its relatively high abundance. Experimental results showed that MELTF-AS1 could increase metastasis ability of osteosarcoma cells *in vivo* and *in vitro*, and its expression was related to the prognosis of patients with osteosarcoma. More recently, the competitive endogenous RNA (ceRNA) hypothesis was proposed and widely verified. In brief, some RNA transcripts such as mRNAs and lncRNAs competitively bound to the same microRNA (miRNA; a small single-stranded non-coding RNA molecule that functions in RNA silencing and post-transcriptional regulation of gene expression), and they could communicate and regulate each other's expression levels through the shared miRNAs. Here, we found that MELTF-AS1 competed with MMP14, a key pro-metastasis gene, for miR-485-5p and relieved the inhibitory effect of miR-485-5p on MMP14, thereby leading to increased MMP14 expression and metastasis ability of osteosarcoma cells. Thus, the

Received 7 February 2021; accepted 20 August 2021;
https://doi.org/10.1016/j.omtn.2021.08.022.

[5]These authors contributed equally

Correspondence: Shanyong Zhang, MD, PhD, Department of Spine Surgery, The Second Hospital of Jilin University, Changchun 130000, Jilin, China.
E-mail: esucai@jlu.edu.cn

This is an open access article under the CC BY license (http://creativecommons.org/licenses/by/4.0/).

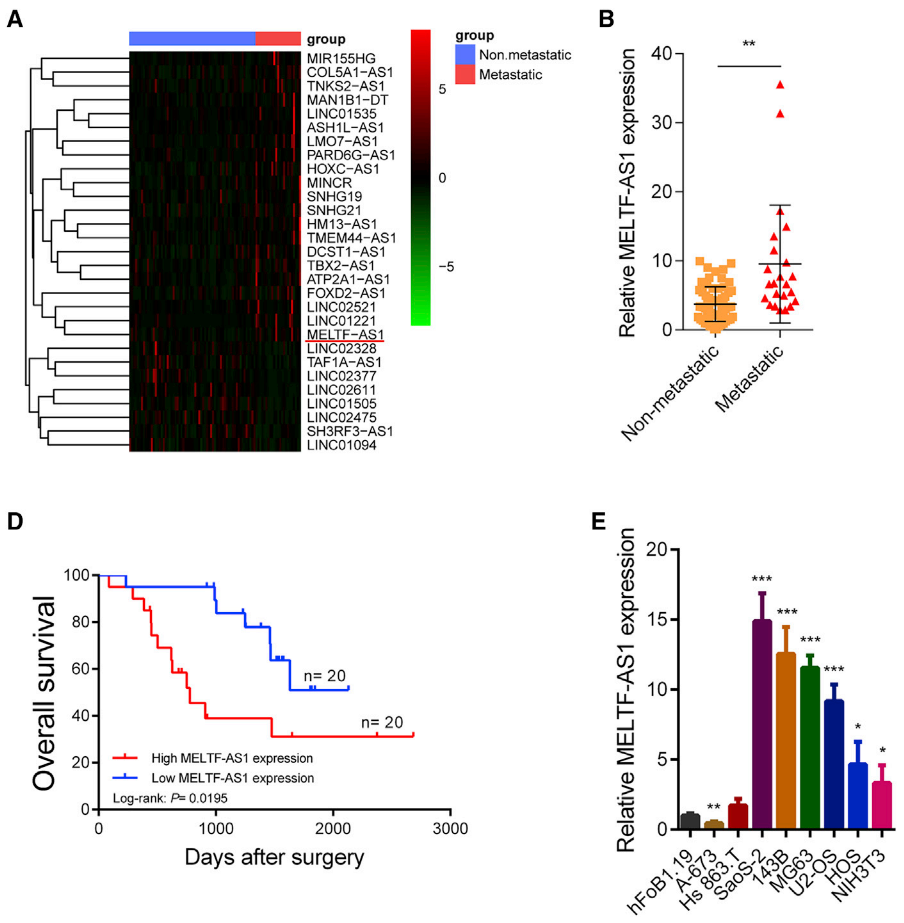

Figure 1. MELTF-AS1 is upregulated in osteosarcoma and is associated with distant metastasis of osteosarcoma

(A) Heatmap of lncRNAs differentially expressed in osteosarcoma with or without distant metastasis. Red indicates upregulation and green indicates downregulation. The red underline denotes MELTF-AS1. (B) The comparison of MELTF-AS1 expression between osteosarcoma tissues with or without distant metastasis in the TARGET database. (C) MELTF-AS1 expression in 40 pairs of osteosarcoma and corresponding normal tissues detected by qRT-PCR. (D) Kaplan-Meier survival analysis of osteosarcoma patients' overall survival based on MELTF-AS1 expression. The median level of MELTF-AS1 is used as the cutoff (n = 40, p = 0.0195). (E) MELTF-AS1 expression in osteosarcoma cell lines (SaoS-2, 143B, MG63, U2-OS, HOS, and NIH 3T3), Ewing's sarcoma cell lines (A-673 and Hs863.T), and osteoblast cells hFoB1.19 detected by qRT-PCR. *p < 0.05; **p < 0.01; ***p < 0.001.

MELTF-AS1/miR-485-5p1/MMP14 axis may provide a novel therapeutic target for osteosarcoma treatment.

RESULTS

MELTF-AS1 is upregulated in osteosarcoma and is associated with distant metastasis of osteosarcoma

By analyzing the transcriptome data of osteosarcoma tissues in the TARGET database (https://ocg.cancer.gov/programs/target), we first identified lncRNAs that were abnormally expressed in osteosarcoma tissues with distant metastasis (Figure 1A). The qRT-PCR results showed that compared with normal tissues, the expression of MELTF-AS1 was significantly higher in osteosarcoma tissues. In addition, consistent with the sequencing results, the expression level of MELTF-AS1 in osteosarcoma tissues with distant metastasis was significantly higher than that in osteosarcoma tissues without metastasis (Figures 1B and 1C). Besides, osteosarcoma patients with high MELTF-AS1 expression have a poor prognosis (Figure 1D). The qRT-PCR analysis of the cell lines showed that the expression of MELTF-AS1 in osteosarcoma cells (SaoS-2, 143B, MG63, U2-OS, HOS, and NIH 3T3) was significantly higher than that in osteoblast cells hFoB1.19. In addition, the expression level of MELTF-AS1 in two Ewing's sarcoma cell lines (A-673 and Hs863.T) was not significantly higher than that of hFoB1.19, indicating that MELTF-AS1 may only play a specific role in osteosarcoma (Figure 1E).

RREB1 activates the transcription of MELTF-AS1 in osteosarcoma

Next, we explored why the expression of MELTF-AS1 was elevated in osteosarcoma. We used the JASPAR database[10] to predict transcription factors that might bind to the promoter region of MELTF-AS1.

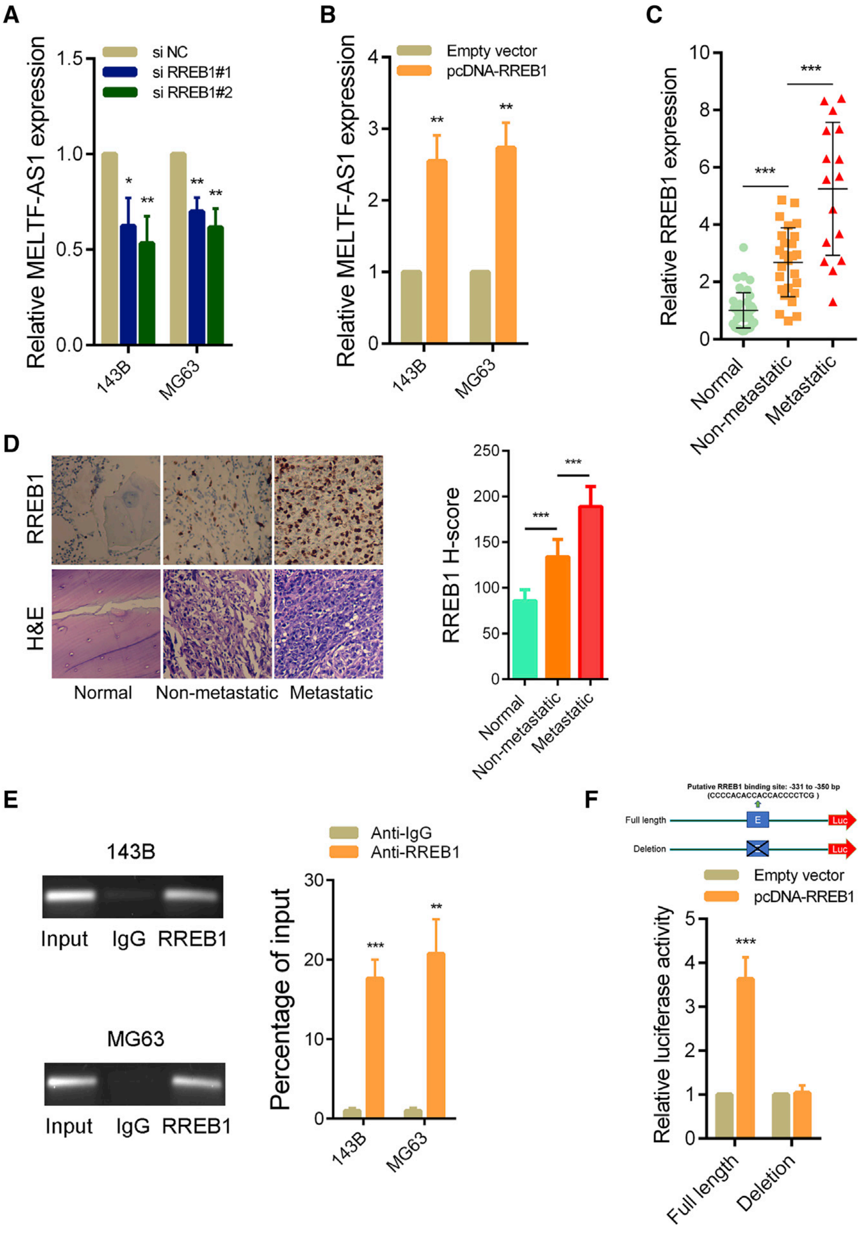

Figure 2. RREB1 binds to the promoter region of MELTF-AS1 and activates its transcription in MELTF-AS1

(A and B) qRT-PCR analysis of MELTF-AS1 expression in 143B and MG63 cells following RREB1 knockdown and overexpression. (C) qRT-PCR analysis of RREB1 expression in 40 pairs osteosarcoma tissues and corresponding adjacent normal tissues. (D) *In situ* hybridization (ISH) analysis of RREB1 expression in 40 pairs osteosarcoma tissues and corresponding adjacent normal tissues. (E) ChIP-qRT-PCR analysis of RREB1 occupancy in the MELTF-AS1 promoter in 143B and MG63 cells. (F) Luciferase reporter assays of the 143B cells transfected with reporter vector containing full length of the MELTF-AS1 promoter or containing the promoter with deletion of binding sites, the pcDNA-RREB1 vector, or an empty vector. *$p < 0.05$; **$p < 0.01$; ***$p < 0.001$.

Among these candidate transcription factors, RREB1 obtained a relatively high score (Table S1). After silencing RREB1, the expression level of MELTF-AS1 in osteosarcoma cells was significantly reduced (Figure 2A; Figure S1A), and after overexpression of RREB1, the expression level of MELTF-AS1 increased significantly (Figure 2B; Figure S1B). Furthermore, the results of PCR assays revealed RREB1 was significantly elevated in osteosarcoma tissues, and the expression level in osteosarcoma tissues with distal metastasis was higher than that in osteosarcoma tissues without metastasis (Figure 2C). The results of immunohistochemistry (IHC) assays showed that the trend of RREB1 protein level in osteosarcoma tissues was consistent with the RNA level (Figure 2D). In addition, chromatin immunoprecipitation (ChIP) experiments with the RREB1 antibody showed that it could directly bind to the promoter region of MELTF-AS1 (Figure 2E). In addition, we constructed a luciferase reporter containing the promoter region of MELTF-AS1. When RREB1 was overexpressed, the relative luciferase activity raised significantly, but after the binding region of RREB1 in the promoter was deleted, overexpression of RREB1 did not cause luciferase activity change (Figure 2F).

MELTF-AS1 promotes metastasis of osteosarcoma cells both *in vitro* and *in vivo*

Transwell assays showed that after knocking down MELTF-AS1, the migration and invasion ability of osteosarcoma cells decreased significantly (Figure 3A). The results of scratch wound healing assays also revealed that after MELTF-AS1 was silenced, the migration ability of the osteosarcoma cells decreased significantly (Figure 3B). In addition, the results of tail vein injection lung metastasis model indicated that after inhibiting MELTF-AS1, the metastasis ability of osteosarcoma cells *in vivo* was weakened. As shown in Figure 3C (left panel), the number of pulmonary metastasis nodules formed by osteosarcoma cells in the MELTF-AS1-silenced group (sh MELTF-AS1) was significantly reduced. Also, the survival time of mice in the sh MELTF-AS1 group was longer than that in the control group (Figure 3C, right panel). Immunofluorescence assays revealed that after silencing MELTF-AS1 in osteosarcoma cells, the protein level of Vimentin decreased and the protein level of E-cadherin increased (Figure 3D). Vimentin is highly expressed in mesenchymal cells and is positively correlated with increased metastasis. E-Cadherin is highly expressed in epithelial cells and is correlated with

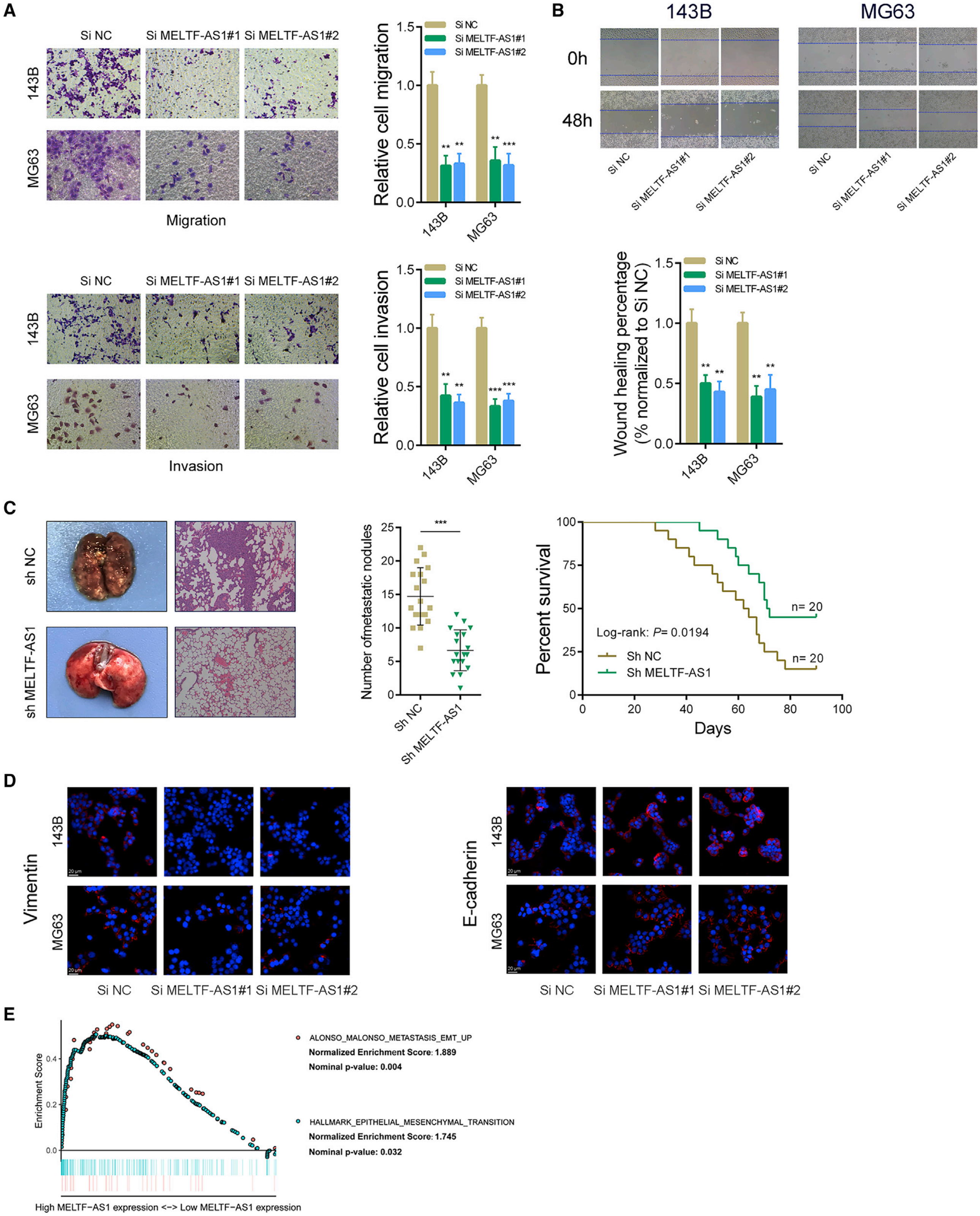

(legend on next page)

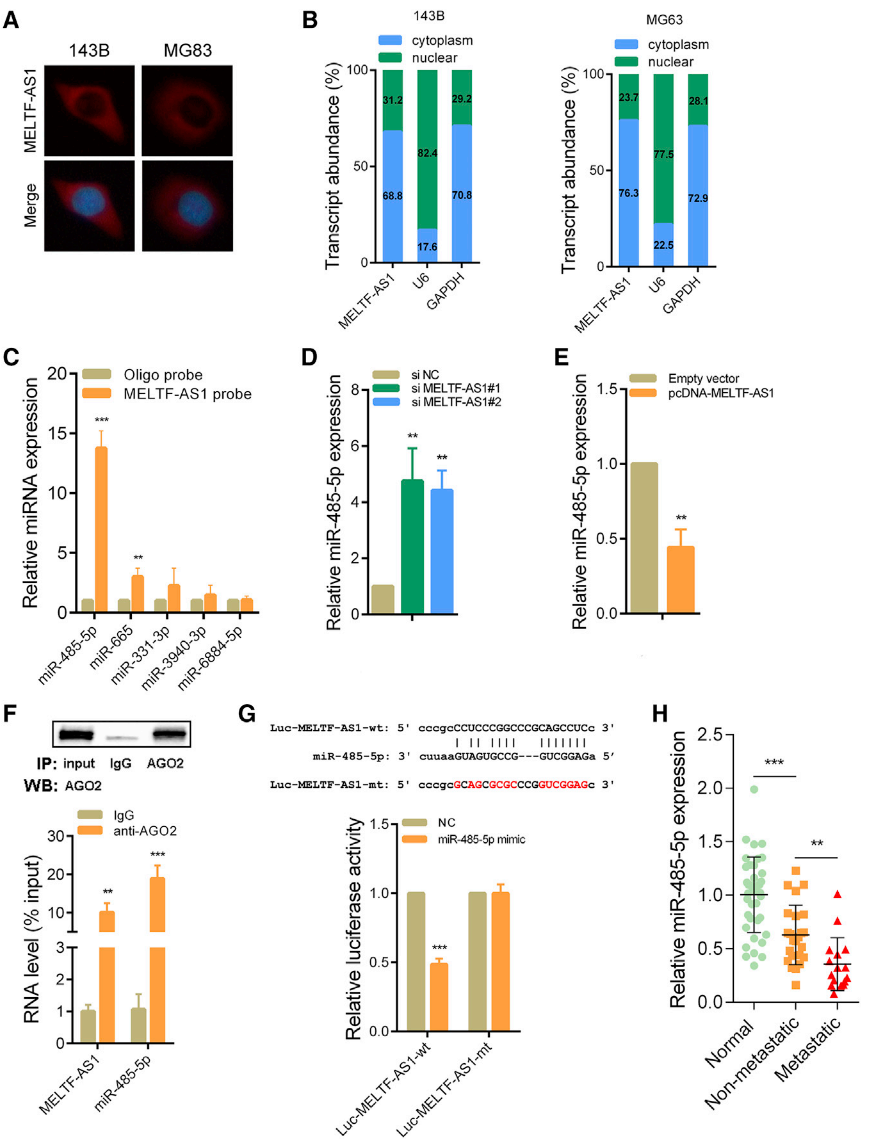

Figure 4. miR-485-5p is a target of MELTF-AS1

(A) Subcellular localization of MELTF-AS1 was observed by RNA *in situ* hybridization. Red: MELTF-AS1; blue: DAPI. (B) qRT-PCR detection of the percentage of MELTF-AS1 in the nuclear and cytoplasm fractions of 143B and MG63 cells. Nuclear controls: U6; cytosolic controls: GAPDH. (C) Interaction between MELTF-AS1 and potential target microRNAs was confirmed by RNA pull-down assays. (D) miR-485-5p expression in 143B cells transfected with MELTF-AS1 siRNAs was detected by qRT-PCR. (E) miR-485-5p expression in 143B cells transfected with pcDNA-MELTF-AS1 was detected by qRT-PCR. (F) RNA immunoprecipitation assays with an anti-AGo2 antibody to assess endogenous AGo2-binding RNAs. MELTF-AS1 and miR-485-5p was determined by qRT-PCR and is presented as fold enrichment in AGo2 relative to input. (G) Sequence alignment of miR-485-5p with the binding sites in the wild-type and mutant-type regions of MELTF-AS1 luciferase reporter (top panel). The relative luciferase activity was detected in 143B cells after co-transfection with luciferase reporter and miR-485-5p mimic. (H) qRT-PCR analysis of miR-485-5p expression in 40 pairs osteosarcoma tissues and adjacent normal tissues. *p < 0.05; **p < 0.01; ***p < 0.001.

decreased metastasis. They are important markers of epithelial-mesenchymal transition (EMT)[11]. Gene set enrichment analysis (GSEA) showed that high MELTF-AS1 expression in osteosarcoma tissues was related to the ALONSO_METASTASIS_EMT_UP and HALLMARK_EPITHELIAL_MESENCHYMAL_TRANSITION gene sets (Figure 3E). These above-mentioned results indicated that MELTF-AS1 could promote metastasis of osteosarcoma cells both *in vivo* and *in vitro* and could affect the metastasis-related pathways in osteosarcoma.

MELTF-AS1 acts as a ceRNA of miR-485-5p in osteosarcoma

Fluorescence *in situ* hybridization (FISH) assays showed that MELTF-AS1 was mainly distributed in cytoplasm of osteosarcoma cells (Figure 4A). qRT-PCR analysis of subcellular fractionation also revealed that MELTF-AS1 was mainly located in the cytoplasm (Figure 4B). Considering that lncRNAs in cytoplasm can function as ceRNAs, we used the ENCORI database[12] to predict potential bindings of microRNAs to MELTF-AS1. Next, we verified which microRNAs could bind to MELTF-AS1 by the RNA pull-down assays with biotin-labeled MELTF-AS1 probe. As shown in Figure 4C, miR-485-5p and miR-665 could bind to MELTF-AS1 directly. Because the binding abundance of miR-485-5p and MELTF-AS1 is higher, we chose it for further study. miR-485-5p expression was significantly increased after MELTF-AS1 silencing in 143B cells (Figure 4D; Figure S2A). Conversely, miR-485-5p expression was significantly

Figure 3. Knockdown of MELTF-AS1 inhibits osteosarcoma metastasis both *in vitro* and *in vivo*

(A) The migration and invasion ability of 143B and MG63 cells was assessed using transwell assays after silencing MELTF-AS1. (B) The migration ability was detected in 143B and MG63 cells after silencing MELTF-AS1 by wound healing assays. (C) Representative images of lungs from mice after tail vein injections with stably transfected MELTF-AS1 shRNAs or sh-negative control (sh-NC) 143B cells (left). Quantitative analysis of metastasis foci in corresponding groups (middle). Survival analysis of mice after tail vein injections with stably transfected sh-MELTF-AS1 and sh-NC cells (right). (D) Vimentin and E-cadherin expression was detected in 143B and MG63 cells after transfected with MELTF-AS1 siRNAs by immunofluorescence assays. (E) The GSEA results were plotted to visualize the correlation between the expression of MELTF-AS1 and the gene sets related to cancer metastasis (ALONSO_METASTASIS_EMT_UP, HALLMARK_EPITHELIAL_MESENCHYMAL_TRANSITION). *p < 0.05; **p < 0.01; ***p < 0.001.

reduced when MELTF-AS1 was overexpressed (Figure 4E; Figure S2B). The results of the RNA immunoprecipitation experiments revealed that both MELTF-AS1 and miR-485-5p could directly bind to Argonaute 2 (AGO2) protein (Figure 4F). The AGO2 is an effector protein partner of microRNAs in the cytoplasmic RNA-induced silencing complex.[13] In addition, we constructed a dual luciferase reporter (Luc-MELTF-AS1-wt) based on the binding sites of MELTF-AS1 and miR-485-5p. Experimental results showed that overexpression of miR-485-5p could significantly reduce the luciferase activity. However, if the binding sites of MELTF-AS1 and miR-485-5p were mutated (Luc-MELTF-AS1-mt), overexpression of miR-485-5p had almost no effect on luciferase activity (Figure 4G). In addition, the qRT-PCR analysis of osteosarcoma tissues showed that the expression of miR-485-5p was reduced in osteosarcoma, and the expression of miR-485-5p was lower in osteosarcoma tissues with distant metastasis (Figure 4H).

MELTF-AS1 regulates MMP14 expression by interaction with miR-485-5p

We then used bioinformatics tools to predict target genes of miR-485-5p, and the results showed that the metastasis-related protein MMP14 was a potential target of miR-485-5p. The results of the luciferase reporter assays revealed that miR-485-5p could directly bind to the 3′ UTR of MMP14 and cause a decrease in luciferase activity. When the binding site was mutated, miR-485-5p had no effect on luciferase activity (Figure 5A). qRT-PCR results showed that the RNA level of MMP14 decreased after MELTF-AS1 was silenced in osteosarcoma cells, and the decreased MMP14 returned to normal level when silencing MELTF-AS1 was combined with transfection of miR-485-5p inhibitors (Figure 5B). In addition, overexpression of MELTF-AS1 in osteosarcoma cells could lead to increased expression of MMP14, but if overexpression of MELTF-AS1 was accompanied by overexpression of miR-485-5p, the increase of MMP14 was weakened (Figure 5C). The western blot results showed that the changes in protein levels were consistent with the changes in RNA levels (Figure 5D). In addition, RNA level of MMP14 was elevated in osteosarcoma tissues, and it was higher in osteosarcoma tissues with distant metastasis than in non-metastatic osteosarcoma tissues (Figure 5E). Correlation analysis in osteosarcoma tissues indicated that the expression of miR-485-5p was negatively correlated with the expression of MELTF-AS1 and MMP14, while the expression of MELTF-AS1 was positively correlated with the expression of MMP14 (Figure 5F). In summary, MELTF-AS1 adsorbs miR-485-5p in cytoplasm and acts as a ceRNA to promote MMP14 expression.

MELTF-AS1 promotes osteosarcoma metastasis by regulating MMP14

The results of the transwell experiments showed that overexpression of MELTF-AS1 increased migration and invasion abilities of osteosarcoma cells. Overexpression of MELTF-AS1, while overexpressing miR-485-5p or inhibiting MMP14, impaired the increase in metastasis ability caused by MELTF-AS1 overexpression (Figure 6A). Immunofluorescence assays demonstrated that overexpression of MELTF-AS1 increased the expression of Vimentin and decreased the expression of E-cadherin, and co-transfection of miR-485-5p or MMP14 siRNAs eliminated the changes caused by MELTF-AS1 (Figure 6B). In addition, western blot revealed that MELTF-AS1 overexpression promoted the expression of VEGF-A, a downstream target of MMP14. Similarly, overexpression of miR-485-5p or inhibition of MMP14 attenuated the elevation of VEGF-A induced by MELTF-AS1 (Figure 6C). These above-mentioned results indicate that MELTF-AS1 affects metastasis-related pathways and proteins by regulating MMP14 and promotes osteosarcoma cells metastasis (Figure 7).

DISCUSSION

In recent years, the role of lncRNAs in osteosarcoma has attracted much attention. Many lncRNAs have been revealed to play important roles in osteosarcoma. For instance, Zhu et al.[14] reported that lncRNA ODRUL contributed to osteosarcoma progression through increasing MMP2. Zhao et al.[15] demonstrated that exosome-derived lncRNA PVT1 promoted osteosarcoma growth and metastasis by stabilizing ERG. Shen et al.[16] found that KCNQ1OT1 promotes osteosarcoma growth by enhancing aerobic glycolysis. However, the role of many lncRNAs in osteosarcoma is still unclear; thus, further exploration is needed.

Osteosarcoma is a highly aggressive cancer common in children and young adults. The therapeutic effect of osteosarcoma has made great progress in the past few decades, but the effective treatment of metastatic or recurrent osteosarcoma is still a clinical problem that needs to be solved urgently. Despite intensive efforts, few novel regimens have been developed to improve survival of metastatic patients.[4,17,18] Elucidating the molecular mechanism of osteosarcoma metastasis may provide new ideas and targets for treatment of metastatic osteosarcoma. Here, we focused on exploring the roles of lncRNAs in osteosarcoma metastasis. We first identified abnormally expressed lncRNAs in metastatic osteosarcoma by analyzing high-throughput transcriptome data of osteosarcoma tissues. From these metastasis-related lncRNAs, we selected MELTF-AS1 for further study. The reasons for choosing MELTF-AS1 for further research are as follows: (1) the abundance of MELT-AS1 in these deregulated lncRNAs is relatively high; (2) the expression level of MELTF-AS1 is related to the prognosis of patients with osteosarcoma; and (3) the role of MELTF-AS1 in osteosarcoma has not been reported before. MELTF-AS1 is located in the Q29 region of chromosome 3. Currently, there are few studies on its function. Flippot et al.[19] reported that MELTF-AS1 expression could be used as a biomarker to predict the recurrence of renal clear cell carcinoma. Luo et al.[20] reported that MELTF-AS1 was involved in the pathological process of osteoarthritis induced by lipopolysaccharide (LPS) through regulating TCF4 expression. Our study is the first to explore its role in osteosarcoma. We detected MELTF-AS1 expression in 40 pairs of osteosarcomas and paracancerous tissues and found that MELTF-AS1 expression was elevated in osteosarcoma tissues compared with paracancerous tissues and that MELTF-AS1 expression was higher in metastatic osteosarcoma than in non-metastatic osteosarcoma. Furthermore, high expression of MELTF-AS1 predicted a poor

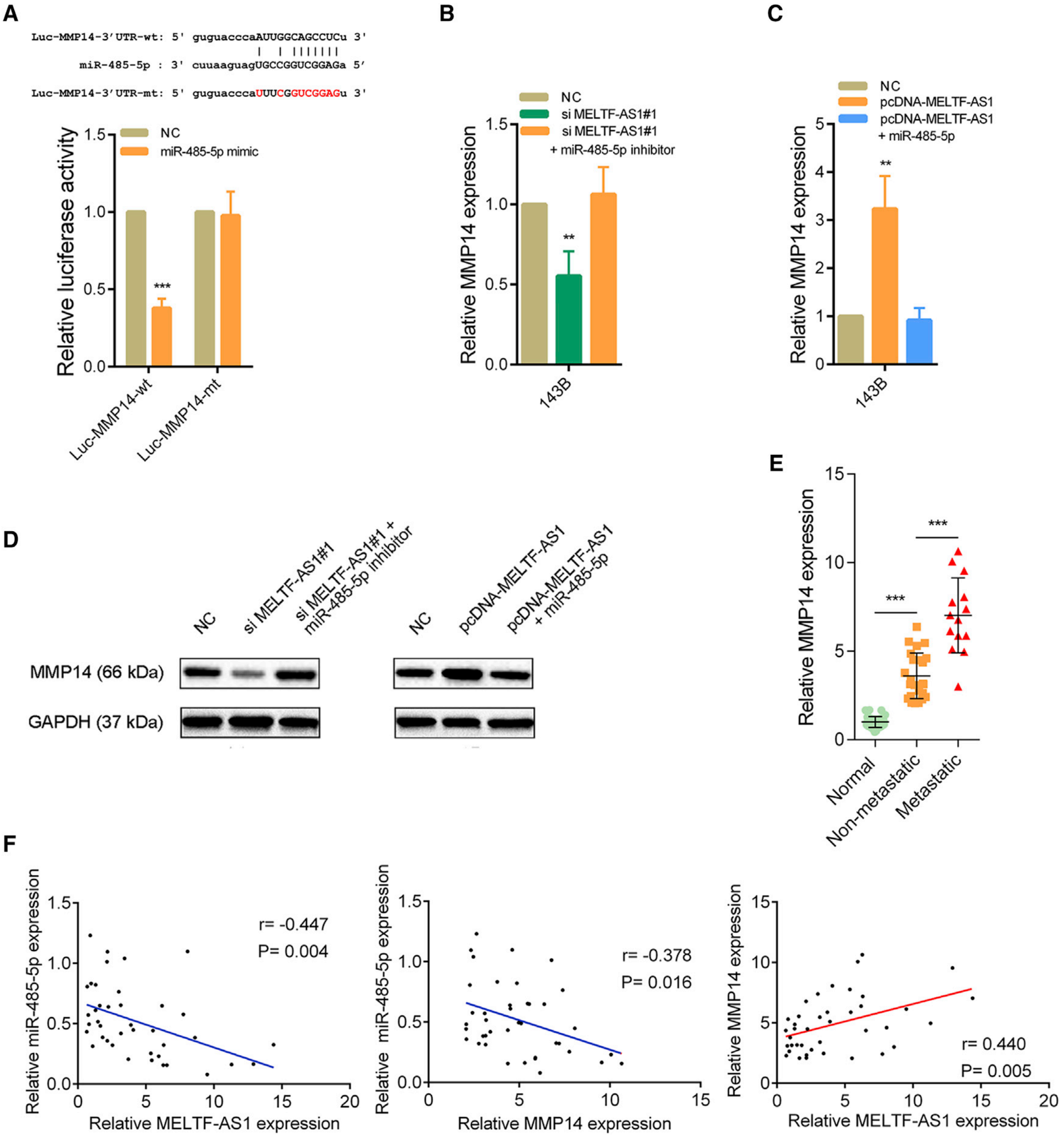

Figure 5. MELTF-AS1 promotes MMP14 expression through adsorbing miR-485-5p

(A) Dual luciferase reporter assays were conducted in the 143B cells co-transfected with the miR-485-5p mimics and luciferase reporter plasmids that were inserted with the wild-type MMP14 3′ UTR or binding sites-mutated MMP14 3′ UTR. (B) qRT-PCR analysis of MMP14 expression after knockdown of MELTF-AS1 or knockdown of MELTF-AS1 combining inhibition of miR-485-5p in 143B cells. (C) qRT-PCR analysis of MMP14 expression after overexpressing MELTF-AS1 or overexpressing MELTF-AS1 combining overexpressing miR-485-5p in 143B cells. (D) MMP14 expression was detected by western blot in 143B cells with indicated treatments. (E) qRT-PCR analysis of MMP14 expression in 40 pairs osteosarcoma tissues and corresponding adjacent normal tissues. (F) Correlation analysis between MELTF-AS1 and miR-485-5p (r = 0.-447, p = 0.004), MMP14 and miR-485-5p (r = −0.378, p = 0.016), and MELTF-AS1 and MMP14 (r = 0.440, p = 0.005) in 40 paired osteosarcoma samples. **p < 0.01; ***p < 0.001.

prognosis of osteosarcoma patients, possibly because osteosarcoma tissues with distal metastasis tend to have higher MELTF-AS1 expression. In addition, both *in vivo* and *in vitro* functional experiments have shown that MELTF-AS1 knockdown reduced the metastatic ability of osteosarcoma cells. However, our study did not observe the effect of knockdown MELTF-AS1 on the growth of osteosarcoma cells. The role of MELTF-AS1 in the growth of osteosarcoma needs to be further explored. MELTF-AS1 is mainly located in the cytoplasm. Previous studies have shown that lncRNAs in the cytoplasm can regulate expression of target genes by absorbing microRNAs.[21,22]

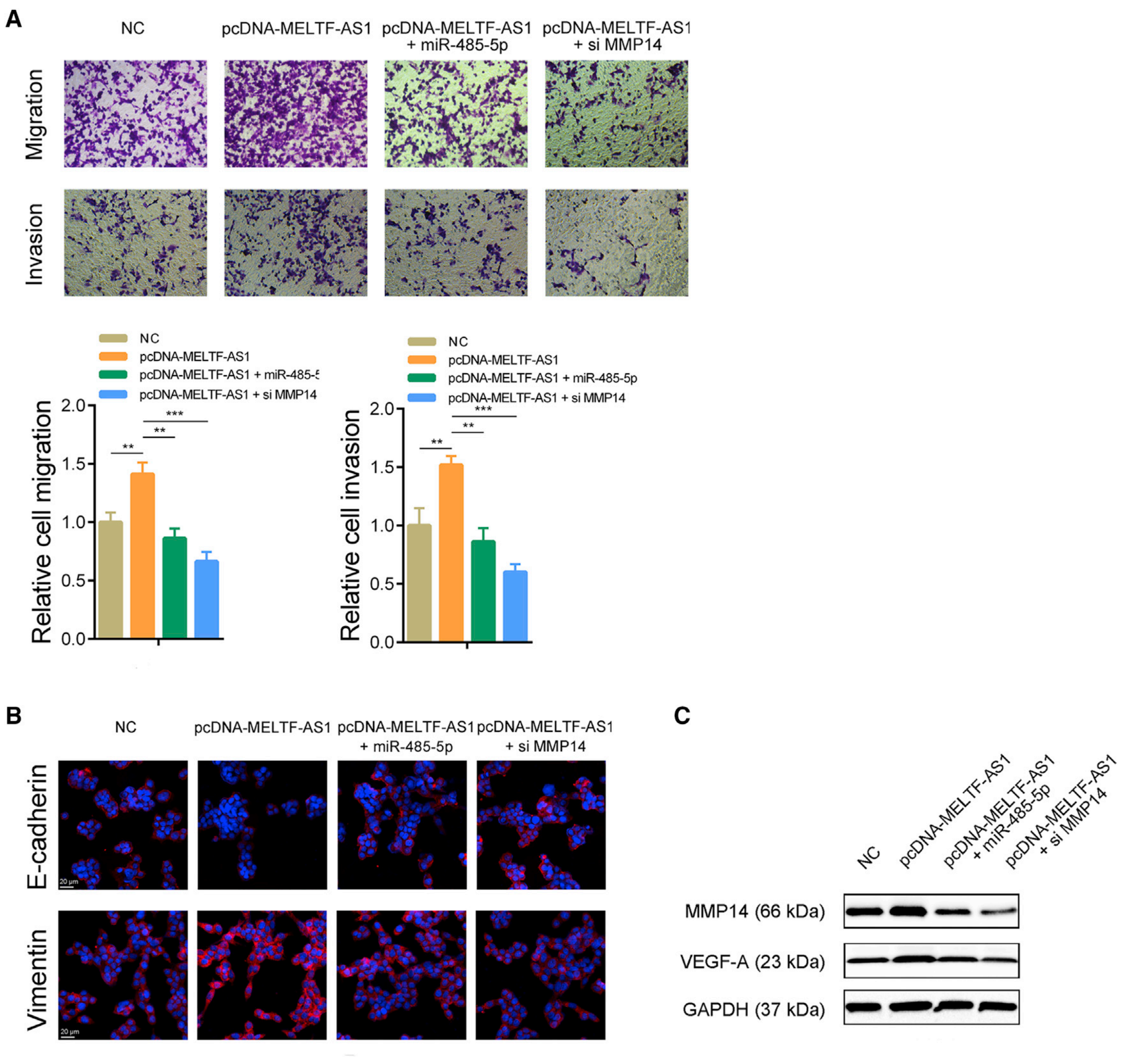

Figure 6. MELTF-AS1 exhibits pro-metastasis property partly dependent on MMP14

(A) Transwell assays were conducted to determine migration and invasion abilities of 143B cells with indicated treatment. (B) Vimentin and E-cadherin expression was detected by immunofluorescence assays in 143B cells with indicated treatment. (C) MMP14 and VEGF-A expression was detected by western blot in 143B cells with indicated treatment. $^{**}p < 0.01$; $^{***}p < 0.001$.

Our results revealed that MELTF-AS1 could act as a ceRNA to regulate MMP14 expression through interacting with miR-485-5p. miR-485-5p is a widely reported tumor suppressor. Lou et al.[23] reported that miR-485-5p suppressed metastasis of breast cancer cells by inhibiting PGC-1α expression. Chen et al.[24] reported that miR-485-5p inhibited bladder cancer metastasis by targeting HMGA2. Kang et al.[25] found that miR-485-5p acted as a negative regulator in gastric cancer progression by targeting flotillin-1. Here, we found that the expression of miR-485-5p was reduced in osteosarcoma and lower in metastatic osteosarcoma. The results also showed that miR-485-5p could interact with both MELTF-AS1 and MMP14. MMP14 is a membrane-anchored matrix metalloproteinase and has been shown to be a key player in both extracellular matrix remodeling and cell migration during cancer metastasis.[26–28] We found that MMP14 expression was significantly increased in osteosarcoma tissues with distant metastasis. Moreover, the enhanced metastatic ability of MELTF-AS1 was partly dependent on its promotion of the expression of MMP14 and its downstream gene VEGF-A.[29] Some recent studies have shown that small interfering RNAs (siRNAs) targeting onco-lncRNAs can be packaged into nanoparticles as drugs to treat tumors.[30,31] Further studies are needed to explore whether MELTF-AS1-specific siRNAs or other inhibitors can be used as potential drugs for osteosarcoma.

In conclusion, MELTF-AS1 is a pro-metastatic lncRNA in osteosarcoma. MELTF-AS1 expression is elevated in osteosarcomas with distant metastasis, and its high expression was associated with poor prognosis of osteosarcoma patients. MELTF-AS1 can enhance the

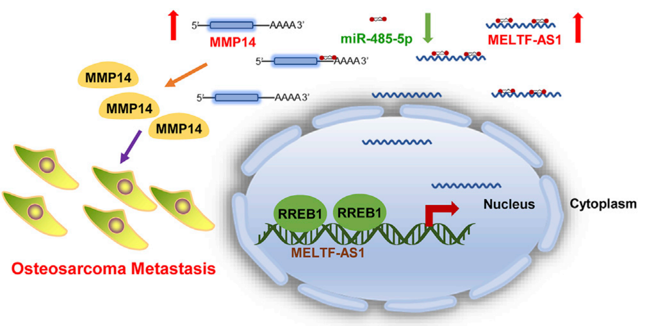

Figure 7. Schematic representation of the potential molecular mechanisms of MELTF-AS1 involved in osteosarcoma

Mechanistically, RREB1 activates MELTF-AS1 transcription in osteosarcoma. MELTF-AS1 competes with MMP14, a key pro-metastasis gene, for miR-485-5p and relieves the inhibitory effect of miR-485-5p on MMP14, thereby leading to increased MMP14 expression and metastasis ability of osteosarcoma cells.

metastatic ability of osteosarcoma cells by regulating MMP14 expression. Our research indicates that MELTF-AS1 may be a potential therapeutic target for patients with osteosarcoma.

MATERIALS AND METHODS

Clinical samples and clinical data collection

Osteosarcoma and paired noncancerous tissues were collected from patients who underwent resection at The Second Hospital of Jilin University between June 2013 and March 2020. None of the enrolled patients had received any chemotherapy or radiotherapy prior to surgery. Tissue samples were snap-frozen in liquid nitrogen and stored at −80 °C until RNA extraction. Written informed consent for research was given by all the patients. This study was approved by the Ethics Committee of Jilin University.

Cell culture

The human osteosarcoma cell lines SaoS-2, MG63, U2-OS, and HOS and Ewing's sarcoma cell lines A-673 and Hs863.T cells were purchased from the Procell Life Science and Technology, China. The human osteosarcoma cell line 143B and the human osteoblast cells hFoB1.19 were purchased from the ATCC, USA. These cells were cultured at 37°C with 5% CO_2 according to the standard protocols. SaoS-2 and U2-OS cells were cultured in McCoy's 5A medium supplemented with 10% fetal bovine serum (FBS) and antibiotics (penicillin, 100 U/mL; streptomycin, 0.1 mg/mL). MG63, HOS, and 143B cells were cultured in Eagle's minimal essential medium (EMEM) with 10% FBS and antibiotics. The hFoB1.19, A-673, and Hs863.T cells were cultured in Gibco Dulbecco's modified Eagle's medium: Nutrient Mixture F-12 (DMEM/F-12) medium with 10% FBS and antibiotics.

RNA isolation and quantitative real-time PCR

Total RNA was isolated from tissues and cells using TRIzol reagents (Thermo Fisher Scientific, USA) according to the standard protocol. microRNA cDNA was synthesized using the microRNA 1st Strand cDNA Synthesis Kit (Vazyme, China). lncRNA and mRNA cDNA was synthesized using the SuperScript IV First-Strand Synthesis System (Thermo Fisher Scientific, USA). qRT-PCR was performed with SYBR Green Master Mix (Thermo Fisher Scientific, USA). The following primer sequences were used for qRT-PCR: for MELTF-AS1, 5′-GCGTTCACACTCATTACCC-3′ (forward) and 5′-CTATTCAGACCCCTTCACCC-3′ (reverse); for RREB1, 5′-AGGTTCAGACCTATCTTCCATCA-3′ (forward) and 5′-CTGCCAATCCGATTTGGTCCT-3′ (reverse); for MMP14, 5′-CGAGGTGCCCTATGCCTAC-3′ (forward) and 5′-CTCGGCAGAGTCAAAGTGG-3′ (reverse); and for GAPDH, 5′-GGAGCGAGATCCCTCCAAAAT-3′ (forward) and 5′-GGCTGTTGTCATACTTCTCATGG-3′ (reverse).

Western blot

Western blot was performed according to the standard protocol. Briefly, proteins were segregated on SDS-polyacrylamide gels and transmitted to polyvinylidene fluoride (PVDF) membranes. The membranes were then incubated with specific primary antibodies: RREB1 antibody (#NBP1-71884, Novus Biologicals, USA), Argonaute 2 antibody (#ab32381, Abcam, USA), MMP14 antibody (#ab51074, Abcam, USA), VEGF-A antibody (#ab1316, Abcam, USA), and GAPDH antibody (#2118, Cell Signaling Technology, USA) overnight at 4°C. After the membranes were incubated with secondary antibodies, they were subjected to immunoblot analysis using an enhanced chemiluminescence (ECL) immunoblotting kit according to the manufacturer's protocol.

IHC

IHC staining was performed using Dako Envision System (Dako, USA) according to the protocol. The IHC-stained tissue sections were scored by two pathologists. The percentages of immunostaining and the staining intensity (0, negative; 1+, weak; 2+, moderate; and 3+, strong) were recorded. An H-score was calculated using the following formula: [1 × (% cells 1+) + 2 × (% cells 2+) + 3 × (% cells 3+)] × 100.

Plasmid construction and cell transfection

The synthetic cDNA sequences of RREB1 (NM_001003698.4) and MELTF-AS1 (NR_038285.1) were sub-cloned to pcDNA3.1 expression vector. The small hairpin RNA (shRNA) targeting MELTF-AS1 was synthesized and sub-cloned into the pLKO.1 vector. The shRNA plasmids were packaged into virus particles using HEK293T cells. The siRNAs of MELTF-AS1, RREB1, and MMP14 were purchased from Ambion (USA). The mimics and inhibitors of miR-485-5p were purchased from the RiboBio (China). The ectopic expression plasmids and shRNAs were transfected into osteosarcoma cells using Lipofectamine 3000 (Invitrogen, USA) as protocol. siRNAs, mimics, and inhibitors of microRNAs were transfected into osteosarcoma cells using Lipofectamine 2000 (Invitrogen, USA) according to the manufacturer's instructions. To establish stable MELTF-AS1 knockdown cell lines, the 143B cells were infected with pLKO.1-sh-MELTF-AS1 lentivirus and selected in the presence of 2 μg/mL puromycin. The efficiency of the knockdown or overexpression was verified by western blot or qRT-PCR.

ChIP assay

The ChIP assay was performed using the Magna ChIP Kit (Merk-Millipore, USA) according to the manufacturer's instructions. In brief, cells were fixed in formaldehyde and then sonicated to generate DNA fragments of 200–800 bp. ChIP assays were performed with the RREB1 antibody (#ab113287, Abcam, USA), or isotype immunoglobulin G (IgG) antibodies. ChIP primer for the MELTF-AS1 promoter was 5′-GCTGGCTTGTTTGGCTTTATT-3′ (forward) and 5′-CAATCCAGCGTCACTTACACT-3′ (reverse).

Transwell assay

Transwell chambers were purchased from Corning Costar (USA). Briefly, the osteosarcoma cells (5×10^4 for migration, 1×10^5 for invasion) were placed onto the upper compartments of the transwell chambers (pre-coated Matrigel for invasion assay) with 100 μL of serum-free medium. Complete medium with 10% FBS was added to the lower chambers. After 24 h, the cells at the membrane were fixed with methanol for 20 min and stained with crystal violet for 30 min. The migrated cells in chambers were photographed and counted.

RNA pull-down assay

Biotin-labeled anti-sense oligonucleotide probes of MELTF-AS1 were designed and purchased from RiboBio (China). Briefly, 10^7 143B cells in total were washed with PBS and cross-linked with a 1% paraformaldehyde solution. Fixed cells were then lysed with lysis buffer (50 mM Tris-HCl [pH 7.0], 10 mM EDTA, and 1% SDS) and sonicated. Biotinylated oligonucleotide probes of MELTF-AS1 (100 pmol to each sample) were hybridized with lysis in hybridization buffer at room temperature for 4 h. After that, magnetic beads were added and incubated overnight. Purified microRNAs were evaluated by qRT-PCR analysis.

Dual luciferase reporter assay

The MELTF-AS1 full sequence and MELTF-AS1 containing the potential binding site of miR-485-5p-mutated or the MMP14 3′ UTR sequence containing the predicted binding site of miR-485-5p and the mutant sequence were synthesized and sub-cloned into the pmirGLO Vector (Promega, USA). The 143B cells were co-transfected with the luciferase reporters, along with miR-485-5p mimic, or the negative control using Lipofectamine 2000. After 48 h, the relative luciferase activity was measured.

GSEA

The GSEA was conducted to explore pathways that associated with MELTF-AS1 expression in osteosarcoma. Gene expression profiles of 88 osteosarcoma samples were downloaded from the TARGET dataset. According to the MELTF-AS1 expression level order, the top 25% and the bottom 25% of samples were defined as high MELTF-AS1 group and low MELTF-AS1 group, respectively. GSEA v.4.0 was used to determine whether the members of the gene sets from the MSigDB database (H, hallmark gene sets and C2, curated gene sets) were randomly distributed at the top or bottom of the ranking. If most members of a gene set were positively or negatively related to the MELTF-AS1, the set was termed associated with MELTF-AS1.

Tail vein metastasis model

Four-week-old male nude (BALB/c) mice were tail vein injected with 5×10^6 sh-MELTF-AS1 or sh-NC stably transfected 143B cells. Three months later, all mice were killed and the lungs were surgically removed and fixed in 10% neutral phosphate-buffered formalin, followed by H&E stain, and the metastases nodules were counted. Survival analysis was conducted using the Kaplan-Meier method.

Statistical analysis

All statistics were performed using Prism (GraphPad Software, USA) and SPSS (IBM, USA). Differences between the different groups were tested using the two-tailed Student's t test or one-way ANOVA. Kaplan-Meier method was used to evaluate the survival rate and analyzed by log rank test. Data are expressed as the mean ± standard deviation for at least three independent experiments. The correlations were analyzed using Pearson's correlation coefficients. A value of $P < 0.05$ was considered statistically significant.

SUPPLEMENTAL INFORMATION

Supplemental information can be found online at https://doi.org/10.1016/j.omtn.2021.08.022.

ACKNOWLEDGMENTS

The study is supported by the National Natural Science Foundation of China (grant 81902483), the Natural Science Foundation of Jilin Province (grant 20200201563JC), and the Bethune Plan Project of Jilin University (grant 2020B11). We appreciate our colleagues for their valuable efforts and comments on this paper. We appreciate the Therapeutically Applicable Research to Generate Effective Treatments (TARGET) program (https://ocg.cancer.gov/programs/target) for supplying transcriptome data of osteosarcoma.

AUTHOR CONTRIBUTIONS

S.Z., L.D., and T.L. conceived and designed the study. L.D., Y.Q., T.L., L.G., and F.Q. performed the experiments. Z.K., W.G., and Y.Q. collected clinical samples. H.Z. and J.J. analyzed the data. L.D. and T.L. wrote the paper. S.Z. and L.D. revised the paper. All authors read and approved the final manuscript.

DECLARATION OF INTERESTS

The authors declare no competing interests.

REFERENCES

1. Ottaviani, G., and Jaffe, N. (2009). The epidemiology of osteosarcoma. Cancer Treat. Res. *152*, 3–13.
2. Harrison, D.J., Geller, D.S., Gill, J.D., Lewis, V.O., and Gorlick, R. (2018). Current and future therapeutic approaches for osteosarcoma. Expert Rev. Anticancer Ther. *18*, 39–50.
3. Ritter, J., and Bielack, S.S. (2010). Osteosarcoma. Ann. Oncol. *21* (*Suppl 7*), vii320–vii325.
4. Meazza, C., and Scanagatta, P. (2016). Metastatic osteosarcoma: a challenging multidisciplinary treatment. Expert Rev. Anticancer Ther. *16*, 543–556.

5. Iyer, M.K., Niknafs, Y.S., Malik, R., Singhal, U., Sahu, A., Hosono, Y., Barrette, T.R., Prensner, J.R., Evans, J.R., Zhao, S., et al. (2015). The landscape of long noncoding RNAs in the human transcriptome. Nat. Genet. *47*, 199–208.

6. Batista, P.J., and Chang, H.Y. (2013). Long noncoding RNAs: cellular address codes in development and disease. Cell *152*, 1298–1307.

7. Fatica, A., and Bozzoni, I. (2014). Long non-coding RNAs: new players in cell differentiation and development. Nat. Rev. Genet. *15*, 7–21.

8. Huarte, M. (2015). The emerging role of lncRNAs in cancer. Nat. Med. *21*, 1253–1261.

9. Chen, R., Wang, G., Zheng, Y., Hua, Y., and Cai, Z. (2017). Long non-coding RNAs in osteosarcoma. Oncotarget *8*, 20462–20475.

10. Khan, A., Fornes, O., Stigliani, A., Gheorghe, M., Castro-Mondragon, J.A., van der Lee, R., Bessy, A., Chèneby, J., Kulkarni, S.R., Tan, G., et al. (2018). JASPAR 2018: update of the open-access database of transcription factor binding profiles and its web framework. Nucleic Acids Res. *46* (D1), D260–D266.

11. Chaffer, C.L., San Juan, B.P., Lim, E., and Weinberg, R.A. (2016). EMT, cell plasticity and metastasis. Cancer Metastasis Rev. *35*, 645–654.

12. Li, J.H., Liu, S., Zhou, H., Qu, L.H., and Yang, J.H. (2014). starBase v2.0: decoding miRNA-ceRNA, miRNA-ncRNA and protein-RNA interaction networks from large-scale CLIP-Seq data. Nucleic Acids Res. *42*, D92–D97.

13. Ye, Z., Jin, H., and Qian, Q. (2015). Argonaute 2: A Novel Rising Star in Cancer Research. J. Cancer *6*, 877–882.

14. Zhu, K.P., Ma, X.L., and Zhang, C.L. (2017). LncRNA ODRUL Contributes to Osteosarcoma Progression through the miR-3182/MMP2 Axis. Mol. Ther. *25*, 2383–2393.

15. Zhao, W., Qin, P., Zhang, D., Cui, X., Gao, J., Yu, Z., Chai, Y., Wang, J., and Li, J. (2019). Long non-coding RNA PVT1 encapsulated in bone marrow mesenchymal stem cell-derived exosomes promotes osteosarcoma growth and metastasis by stabilizing ERG and sponging miR-183-5p. Aging (Albany NY) *11*, 9581–9596.

16. Shen, Y., Xu, J., Pan, X., Zhang, Y., Weng, Y., Zhou, D., and He, S. (2020). LncRNA KCNQ1OT1 sponges miR-34c-5p to promote osteosarcoma growth via ALDOA enhanced aerobic glycolysis. Cell Death Dis. *11*, 278.

17. Durfee, R.A., Mohammed, M., and Luu, H.H. (2016). Review of Osteosarcoma and Current Management. Rheumatol. Ther. *3*, 221–243.

18. Kager, L., Tamamyan, G., and Bielack, S. (2017). Novel insights and therapeutic interventions for pediatric osteosarcoma. Future Oncol. *13*, 357–368.

19. Flippot, R., Mouawad, R., Spano, J.P., Rouprêt, M., Compérat, E., Bitker, M.O., Parra, J., Vaessen, C., Allanic, F., Manach, Q., et al. (2017). Expression of long non-coding RNA MFI2-AS1 is a strong predictor of recurrence in sporadic localized clear-cell renal cell carcinoma. Sci. Rep. *7*, 8540.

20. Luo, X., Wang, J., Wei, X., Wang, S., and Wang, A. (2020). Knockdown of lncRNA MFI2-AS1 inhibits lipopolysaccharide-induced osteoarthritis progression by miR-130a-3p/TCF4. Life Sci. *240*, 117019.

21. Karreth, F.A., and Pandolfi, P.P. (2013). ceRNA cross-talk in cancer: when ce-bling rivalries go awry. Cancer Discov. *3*, 1113–1121.

22. Salmena, L., Poliseno, L., Tay, Y., Kats, L., and Pandolfi, P.P. (2011). A ceRNA hypothesis: the Rosetta Stone of a hidden RNA language? Cell *146*, 353–358.

23. Lou, C., Xiao, M., Cheng, S., Lu, X., Jia, S., Ren, Y., and Li, Z. (2016). MiR-485-3p and miR-485-5p suppress breast cancer cell metastasis by inhibiting PGC-1α expression. Cell Death Dis. *7*, e2159.

24. Chen, Z., Li, Q., Wang, S., and Zhang, J. (2015). miR-485-5p inhibits bladder cancer metastasis by targeting HMGA2. Int. J. Mol. Med. *36*, 1136–1142.

25. Kang, M., Ren, M.P., Zhao, L., Li, C.P., and Deng, M.M. (2015). miR-485-5p acts as a negative regulator in gastric cancer progression by targeting flotillin-1. Am. J. Transl. Res. *7*, 2212–2222.

26. Poincloux, R., Lizárraga, F., and Chavrier, P. (2009). Matrix invasion by tumour cells: a focus on MT1-MMP trafficking to invadopodia. J. Cell Sci. *122*, 3015–3024.

27. Nguyen, A.T., Chia, J., Ros, M., Hui, K.M., Saltel, F., and Bard, F. (2017). Organelle Specific O-Glycosylation Drives MMP14 Activation, Tumor Growth, and Metastasis. Cancer Cell *32*, 639–653.e6.

28. Zarrabi, K., Dufour, A., Li, J., Kuscu, C., Pulkoski-Gross, A., Zhi, J., Hu, Y., Sampson, N.S., Zucker, S., and Cao, J. (2011). Inhibition of matrix metalloproteinase 14 (MMP-14)-mediated cancer cell migration. J. Biol. Chem. *286*, 33167–33177.

29. Eisenach, P.A., Roghi, C., Fogarasi, M., Murphy, G., and English, W.R. (2010). MT1-MMP regulates VEGF-A expression through a complex with VEGFR-2 and Src. J. Cell Sci. *123*, 4182–4193.

30. Pichler, M., and Rodriguez-Aguayo, C. (2020). Therapeutic potential of FLANC, a novel primate-specific long non-coding RNA in colorectal cancer. Gut *69*, 1818–1831.

31. Bi, Z., Li, Q., Dinglin, X., Xu, Y., You, K., Hong, H., Hu, Q., Zhang, W., Li, C., Tan, Y., et al. (2020). Nanoparticles (NPs)-Meditated LncRNA AFAP1-AS1 Silencing to Block Wnt/β-Catenin Signaling Pathway for Synergistic Reversal of Radioresistance and Effective Cancer Radiotherapy. Adv. Sci. *7*, 2000915.

OMTN, Volume *26*

Supplemental information

lncRNA MELTF-AS1 facilitates osteosarcoma metastasis by modulating MMP14 expression

Lei Ding, Taiyuan Liu, Yuan Qu, Zhichen Kang, Lixin Guo, Haina Zhang, Junjie Jiang, Fuling Qu, Wanbao Ge, and Shanyong Zhang

Supplemental Information

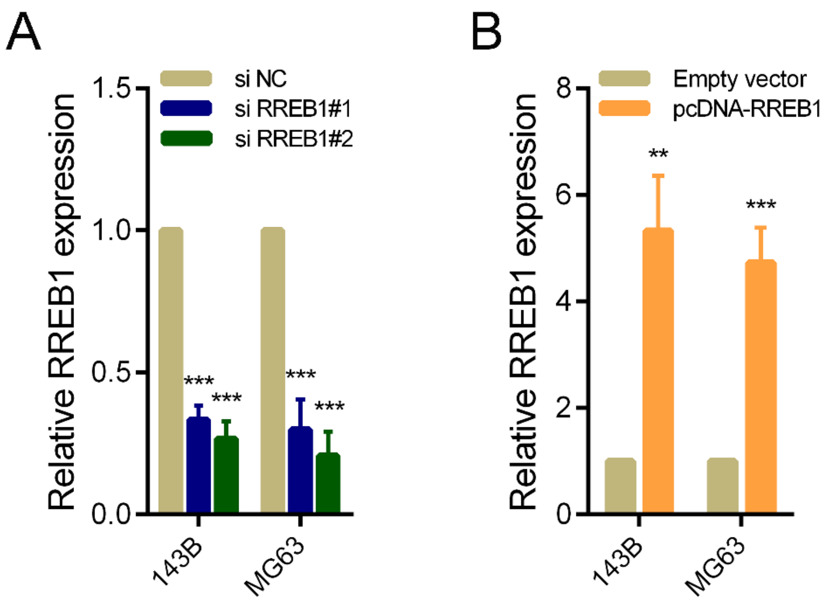

Figure S1. (A) QPCR analysis of RREB1 expression after knockdown of RREB1 in osteosarcoma cells. **(B)** QPCR analysis of RREB1 expression after overexpressing RREB1 in osteosarcoma cells. **P < 0.01 and ***P < 0.001.

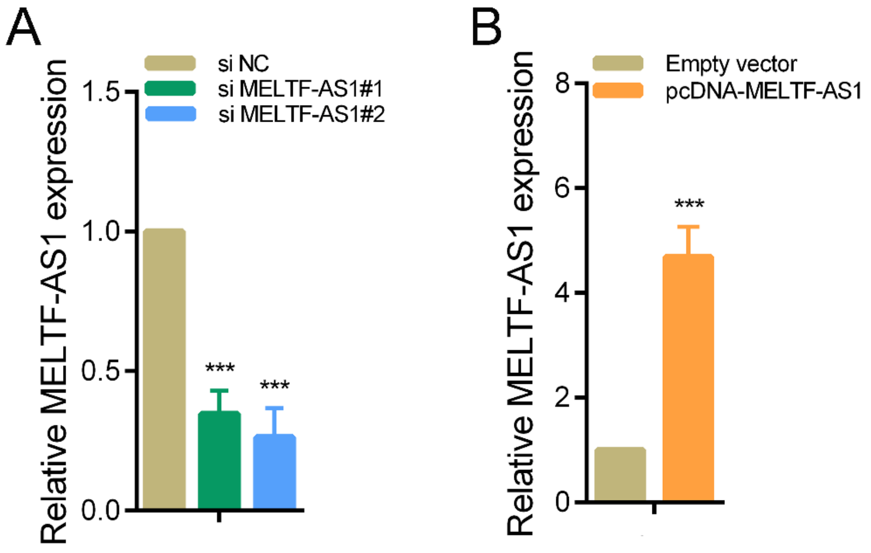

Figure S2. (A) QPCR analysis of MELTF-AS1 expression after knockdown of MELTF-AS1 in 143B cells. **(B)** QPCR analysis of MELTF-AS1 expression after overexpressing MELTF-AS1 in 143B cells. ***P < 0.001.

Table S1: The probability of each transcription factor binding to the MELTF-AS1 promoter region predicted by the JASPAR tool, the higher the score, the greater the probability.

Transcription factor	Score	Start	End	Strand	predicted site sequence
**RREB1**	18.844	312	331	-1	CCCCACACCACCACCCCTCG
**Zfx**	16.178	1491	1504	-1	CCGGCCGGGGCCTC
**E2F3**	16.047	1977	1991	1	CTCCCGCCGCCGCCA
**PPARG::RXRA**	16.039	548	562	1	GTGGGGCAGGGGGCA
**TP63**	15.596	856	875	1	CTGCATGTGCTGGCTTGTTT
**EGR2**	15.411	1721	1735	-1	CCCGCACCCACGCCC
**Spi1**	15.367	1404	1418	-1	ATGAAGAGGAAGAGA
**Zfx**	15.141	1867	1880	1	CCGGGCTCGGCCTC
**EGR1**	15.113	1977	1990	1	CTCCCGCCGCCGCC
**SP2**	14.648	1522	1536	-1	TGCCCGCCCCTCCGC
**EGR1**	14.636	413	426	1	GGCCCACCCCCACC
**ZNF263**	14.560	1394	1414	-1	AGAGGAAGAGAAGAAATGGGA
**E2F3**	14.381	1973	1987	1	CTCCCTCCCGCCGCC
**Myod1**	14.187	631	643	-1	TCCAGCTGCCTCC
**Nkx2-5 (var.2)**	14.170	795	805	-1	AGGCACTCAAA
**HSF1**	13.960	1437	1451	1	GTTCTGGAAACTTTT
**ZNF263**	13.959	1400	1420	-1	AAATGAAGAGGAAGAGAAGAA
**E2F4**	13.950	1975	1985	-1	CGGCGGGAGGG
**RREB1**	13.844	313	332	-1	CCCCCACACCACCACCCCTC
**TP53**	13.843	849	863	-1	ACATGCAGAGGCTTG
**E2F6**	13.669	1876	1886	-1	GGGAGGGAGGC
**E2F6**	13.669	1971	1981	-1	GGGAGGGAGGC
**PRDM1**	13.636	86	100	-1	TGGAAAGGAAAGTTA
**KLF5**	13.540	1915	1924	1	CCCCCACCCC
**Zfx**	13.392	1657	1670	1	GAGCCCGCGGCCTG
**STAT3**	13.347	94	104	-1	CTTCTGGAAAG
**STAT2::STAT1**	13.336	86	100	1	TAACTTTCCTTTCCA
**Myog**	13.329	634	644	1	GGCAGCTGGAA
**TEAD1**	13.305	637	648	-1	CACATTCCAGCT
**POU2F2**	13.293	1337	1349	-1	GCCATTTGCATTC
**RFX2**	13.191	1196	1214	1	GGTTGCATGGCTACAGCCG
**TP53**	13.147	858	872	1	GCATGTGCTGGCTTG
**INSM1**	13.033	551	562	1	GGGCAGGGGGCA
**SP2**	13.021	413	427	1	GGCCCACCCCCACCT
**Tcf12**	12.992	634	644	1	GGCAGCTGGAA
**Zfx**	12.906	1962	1975	1	CCCGGTCCGGCCTC
**Bach1::Mafk**	12.873	912	926	1	GCACTGACACAGCAG
**Nfe2l2**	12.862	911	925	1	AGCACTGACACAGCA
**Spi1**	12.795	108	122	1	AGGGTAGGGAAGTGG
**ZEB1**	12.786	1274	1282	-1	CCCCACCTG
**NR2C2**	12.757	548	562	1	GTGGGGCAGGGGGCA
**ZNF263**	12.735	550	570	1	GGGGCAGGGGGCAGCAGAGAA
**Erg**	12.604	1404	1414	-1	AGAGGAAGAGA
**EGR2**	12.599	1350	1364	1	TCCGCGCCCACGCGG
**Nr1h3::Rxra**	12.591	75	93	1	TGCCCAAAGGTTAACTTTC
**Tcf3**	12.445	148	158	-1	CTCAGCTGCAC
**ESR1**	12.424	1170	1189	-1	AGTCCCGGGCACCTTCACCC
**SP1**	12.418	413	423	1	GGCCCACCCCC
**EGR2**	12.410	413	427	1	GGCCCACCCCCACCT
**INSM1**	12.373	938	949	-1	TGTCAGGTGGCA
**EGR2**	12.343	966	980	1	ACTGCACCCACGCAG
**STAT5**	12.339	248	258	-1	TTTTCCATGAA

**ZNF263**	12.296	1397	1417	-1	TGAAGAGGAAGAGAAGAAATG
**E2F6**	12.239	349	359	1	GAGAGGGAAGC
**Klf1**	12.225	980	990	1	GGCCACACCTT
**Zfx**	12.178	1139	1152	-1	AGGACCTGGGCCTC
**KLF5**	12.124	477	486	1	CCCACGCCCC
**STAT3**	11.996	247	257	1	CTTCATGGAAA
**Sox3**	11.991	1120	1129	-1	GCTTTGTCCC
**TLX1::NFIC**	11.937	1346	1359	1	TGGCTCCGCGCCCA
**SP1**	11.891	1526	1536	-1	TGCCCGCCCCT
**Tcf3**	11.821	633	643	-1	TCCAGCTGCCT
**YY1**	11.821	1475	1486	1	CAAGATGGAGTA
**RUNX2**	11.813	433	447	-1	GTGCTCTGTGGTATC
**RUNX1**	11.773	434	444	-1	CTCTGTGGTAT
**RREB1**	11.722	315	334	-1	GGCCCCCACACCACCACCCC
**EGR2**	11.693	1723	1737	-1	CGCCCGCACCCACGC
**Nfe2l2**	11.682	489	503	1	TCCAGTGAGTTAGCA
**SP1**	11.679	1915	1925	1	CCCCCACCCCG
**Nr2e3**	11.665	1126	1132	-1	CAAGCTT
**E2F1**	11.654	1706	1716	-1	CTGGCGGCAGC
**SREBF2**	11.652	1167	1176	1	GTGGGGTGAA
**E2F6**	11.620	1975	1985	-1	CGGCGGGAGGG
**Sox6**	11.606	1120	1129	-1	GCTTTGTCCC
**Spi1**	11.597	1396	1410	-1	GAAGAGAAGAAATGG
**TP63**	11.594	846	865	-1	GCACATGCAGAGGCTTGGAC
**ELF1**	11.542	1405	1417	-1	TGAAGAGGAAGAG
**EGR1**	11.525	1276	1289	-1	GGCTCGCCCCCACC
**Zfx**	11.509	1219	1232	1	GCTGCCAGGGCCTC
**Klf4**	11.453	1915	1924	-1	GGGGTGGGGG
**PLAG1**	11.434	63	76	1	GAGGGGCTAGGGTG
**RORA_2**	11.416	180	193	1	ACAAAGTCGGTCAA
**TP53**	11.401	859	873	-1	ACAAGCCAGCACATG
**Tcfcp2l1**	11.364	213	226	-1	CCAGTCAATTCCTG
**NFATC2**	11.360	252	258	-1	TTTTCCA
**Tcfcp2l1**	11.316	665	678	1	CCTGCAAGAGCCAG
**SP2**	11.286	1840	1854	-1	GCCCTGCCTCGTCCC
**Ddit3::Cebpa**	11.285	1194	1205	-1	CCATGCAACCCC
**Nfe2l2**	11.279	138	152	1	TGGAGTGACTGTGCA
**NHLH1**	11.214	148	159	-1	TCTCAGCTGCAC
**Klf4**	11.207	477	486	-1	GGGGCGTGGG
**SREBF1**	11.106	1167	1176	-1	TTCACCCCAC
**Tcfcp2l1**	11.099	1812	1825	-1	CCAGCTCCATCCCG
**ESR2**	11.073	1664	1678	-1	AGGTGACCCAGGCCG
**Rfx1**	11.052	1196	1209	1	GGTTGCATGGCTAC
**Zfx**	11.052	1555	1568	-1	ACCCGCTGGGCCTG
**EGR2**	11.024	1561	1575	-1	GCCCCGCACCCGCTG
**USF2**	10.886	938	948	-1	GTCAGGTGGCA
**EGR1**	10.878	1980	1993	1	CCGCCGCCGCCAGC
**Myc**	10.867	858	867	1	GCATGTGCTG
